# TET Enzymes and 5-Hydroxymethylcytosine in Neural Progenitor Cell Biology and Neurodevelopment

**DOI:** 10.3389/fcell.2021.645335

**Published:** 2021-02-18

**Authors:** Ian C. MacArthur, Meelad M. Dawlaty

**Affiliations:** ^1^Department of Genetics, Albert Einstein College of Medicine, Bronx, NY, United States; ^2^Department of Developmental and Molecular Biology, Albert Einstein College of Medicine, Bronx, NY, United States; ^3^Ruth L. and David S. Gottesman Institute for Stem Cell and Regenerative Medicine Research, Albert Einstein College of Medicine, Bronx, NY, United States

**Keywords:** TET enzymes, 5-hydroxymethylcytosine, neural progenitor cells, neurogenesis, neurodevelopmental disorders, epigenetics

## Abstract

Studies of tissue-specific epigenomes have revealed 5-hydroxymethylcytosine (5hmC) to be a highly enriched and dynamic DNA modification in the metazoan nervous system, inspiring interest in the function of this epigenetic mark in neurodevelopment and brain function. 5hmC is generated by oxidation of 5-methylcytosine (5mC), a process catalyzed by the ten–eleven translocation (TET) enzymes. 5hmC serves not only as an intermediate in DNA demethylation but also as a stable epigenetic mark. Here, we review the known functions of 5hmC and TET enzymes in neural progenitor cell biology and embryonic and postnatal neurogenesis. We also discuss how TET enzymes and 5hmC regulate neuronal activity and brain function and highlight their implications in human neurodevelopmental and neurodegenerative disorders. Finally, we present outstanding questions in the field and envision new research directions into the roles of 5hmC and TET enzymes in neurodevelopment.

## Introduction

Precise temporal and spatial control of gene expression is essential for metazoan neurogenesis. This is achieved, in part, by reversible covalent modifications of DNA and histones which influence the accessibility and recruitment of transcription factors to target genes. Methylation of the 5-position carbon of cytosine (5mC) is one DNA modification influencing the transcriptional state of chromatin. DNA methylation is largely believed to be a suppressive mark achieved by *de novo* methyltransferases DNMT3A/B and maintained by maintenance methyltransferase DNMT1 (Wu and Zhang, 2014). In 2009, the discovery that ten-eleven translocation (TET) proteins (TET1, TET2, and TET3) are dioxygenases capable of oxidizing 5mC to 5-hydroxymethylcytosine (5hmC) (Tahiliani et al., 2009) ushered in interest to study this modified base not only as an intermediate in DNA demethylation but also as a novel epigenetic mark. Oxidation of 5mC to 5hmC by TETs facilitates passive and active DNA demethylation (Tahiliani et al., 2009; Ito et al., 2011; Wu and Zhang, 2014), the latter via iterative conversion of 5hmC to 5-formylcytosine (5fC) and 5-carboxylcytosine (5caC) and subsequent removal by DNA glycosylases and the base excision repair pathway (He et al., 2011; Ito et al., 2011). In addition to being an intermediate in DNA demethylation, 5hmC has been recognized as a stable epigenetic mark. This is supported by initial findings that 5hmC is enriched in Purkinje neurons (Kriaucionis and Heintz, 2009), and subsequent studies confirming the presence of 5hmC and the expression of TET enzymes across many neural cell types and tissues (Globisch et al., 2010; Szwagierczak et al., 2010; Ruzov et al., 2011; Mellen et al., 2012). It has been demonstrated that 5hmC may persist for months without turnover in the brain (Bachman et al., 2014), further supporting a potential role for 5hmC as a bona fide epigenetic mark with regulatory roles in the nervous system.

TET enzymes are required for mammalian development, as loss of all three enzymes in embryonic stem cells compromises differentiation (Dawlaty et al., 2014) and in mice leads to early embryonic lethality due to gastrulation arrest (Dai et al., 2016). Loss of TET3 leads to perinatal lethality (Gu et al., 2011), though individual loss of TET1 and TET2 is compatible with development of viable mice (Dawlaty et al., 2011; Li et al., 2011; Moran-Crusio et al., 2011). Combined loss of TET1 and TET2 leads to partial perinatal lethality, with a subset of neonates exhibiting exencephaly and other developmental abnormalities (Dawlaty et al., 2013). Similarly, combined loss of TET1 and TET3 leads to early developmental arrest and holoprosencephaly (Kang et al., 2015). This phenotypic variability suggests potential compensatory roles among TET paralogs. However, owing to the early embryonic lethality of triple TET deficiency, the absolute molecular and physiological requirements of TETs and 5hmC in neurogenesis has not yet been well-defined.

### Genomic Distribution of 5hmC in the Brain

To understand the roles of 5hmC and TETs in regulation of neural gene expression, several studies have mapped the genomic distribution of 5hmC in various neural cell types and tissues over the course of embryonic and postnatal development (Jin et al., 2011; Szulwach et al., 2011; Khare et al., 2012; Hahn et al., 2013; Lister et al., 2013). In the embryonic mouse cortex, 5hmC levels increase as neural progenitor cells develop into mature neurons (Hahn et al., 2013). Interestingly, this increase is not necessarily accompanied by an increase in unmodified cytosine levels, suggesting that 5hmC can be a stable epigenetic mark in neurons and not merely a DNA demethylation intermediate (Hahn et al., 2013). Consistent with the notion that 5hmC is derived from 5mC, genomic regions in the fetal mouse brain that are enriched for 5hmC also tend to be enriched for 5mC. Notably, many of these regions become depleted of both marks in the adult mouse (Lister et al., 2013), demonstrating that 5hmC facilitates DNA demethylation in the developing brain.

5hmC levels increase in various mouse and human brain tissues over the course of life (Szulwach et al., 2011), and may have implications for neurodegenerative diseases. 5hmC is enriched in gene bodies and promoters, depleted from intergenic regions and transcription start sites, and is deposited at brain-specific enhancers (Jin et al., 2011; Szulwach et al., 2011; Hahn et al., 2013; Lister et al., 2013; Cui et al., 2020). The presence of 5hmC in gene bodies is associated with increased gene expression, suggesting that TET enzymes and 5hmC contribute to a transcriptionally permissive state of chromatin in the brain (Jin et al., 2011; Szulwach et al., 2011; Hahn et al., 2013; Lister et al., 2013). 5hmC also demarcates intron-exon boundaries in human brain cells and marks constitutively expressed exons, suggesting a potential role in control of splicing (Khare et al., 2012). Genes with high levels of 5hmC, for example *Syt1* and *Nav2*, belong to functional categories critical for nervous system function, such as synaptic transmission and neurogenesis (Khare et al., 2012; Hahn et al., 2013). 5hmC is also associated with repetitive elements as it is enriched at SINE and LTR elements in the cerebellum and hippocampus, and depleted from LINE elements in the cerebellum (Szulwach et al., 2011). Enrichment at SINE and LTRs increases over postnatal life in the cerebellum (Szulwach et al., 2011), indicating a possible role in regulation of repetitive element activity in the brain. Indeed, *Tet2/3* knockdown reverses loss-of-*Uhrf1*-mediated increased DNA hydroxymethylation and activation of IAP elements in NPCs (Ramesh et al., 2016). Together, these observations support important roles for 5hmC and TETs in mammalian neurogenesis and brain function.

### Regulation of Neural Progenitor Cells and Neurogenesis by TET Enzymes and 5hmC

Studies of embryonic stem cell (ESC) differentiation have suggested a critical role for TET enzymes and 5hmC in neural lineage commitment. Deficiency of all three TETs in ESCs compromises pluripotency and *Tet1/2/3* triple knockout (TKO) ESCs fail to form neural pigmented epithelium in teratoma assays, though they are able to form other neural tissue types (Dawlaty et al., 2014). These cells fail to contribute to nervous system structures when injected into wild type blastocysts to form chimeras (Dawlaty et al., 2014). Consistently, *Tet* TKO mouse embryos and ESCs differentiated toward the neural lineage have reduced neuroectodermal and increased mesodermal gene expression, in part due to failure to inhibit Wnt signaling (Li et al., 2016). Likewise, *TET* TKO human ESCs exhibit aberrant neuroectodermal gene expression when differentiated toward the neural lineage and fail to demethylate the *PAX6* promoter, a transcription factor critical for neurodevelopment (Verma et al., 2018). These studies support a requirement for TET enzymes in the commitment of ESCs to a neural fate, an idea further supported by studies of *TET* genes in ESC specification to neural progenitor cells (NPCs). TET enzymes, in particular TET2, regulate enhancer methylation during differentiation of ESCs to NPCs (Hon et al., 2014). Though *Tet2* knockout ESCs can successfully differentiate into NPCs, these cells exhibit delayed induction of neural gene expression programs accompanied by enhancer hypermethylation and reduced histone H3 lysine 27 acetylation (Hon et al., 2014). This is in line with DNA hypermethylation in the embryonic cerebral cortex of *Tet2* knockout mice (Lister et al., 2013). TET3 plays a role in the epigenetic regulation of NPC specification and maintenance of cellular identity (Montibus et al., 2020; Santiago et al., 2020). During differentiation of mouse ESCs to NPCs, the catalytic activity of TET3 promotes expression of histone demethylase *Kdm6b*, an epigenetic regulator critical for gene regulation during neurogenesis (Montibus et al., 2020), and loss of TET3 promotes NPC apoptosis (Li T. et al., 2015). Knockdown of *Tet3* in NPCs promotes de-repression of pluripotency genes *Oct4* and *Nanog*, implicating TET3 in the maintenance of NPC identity (Santiago et al., 2020). These studies implicate TETs in the epigenetic regulation of NPC biology.

In adult NPCs, different TET paralogs have unique functions, highlighting some non-redundant and context-specific roles. Loss of TET2 increases the proliferation of adult NPCs and reduces their differentiation into neurons and astrocytes *in vivo*, indicating that TET2 promotes NPC differentiation (Li et al., 2017). Deletion of *Tet3* decreases NPC proliferation in the subventricular zone of the mouse cortex and promotes astrocytic differentiation, consistent with a role for TET3 in maintaining NPC identity (Montalban-Loro et al., 2019). *Tet1* knockout mice have fewer NPCs in the dentate gyrus of the hippocampus and conditional deletion of *Tet1* or *Tet2* in NPCs compromises hippocampal neurogenesis (Zhang et al., 2013; Gontier et al., 2018). While most functions of TETs in NPCs are attributed to their enzymatic activity (Zhang et al., 2013; Li et al., 2017; Montibus et al., 2020), some functions are independent of enzymatic activity, such as transcriptional repression of the imprinted gene *Snrnp* by TET3 (Montalban-Loro et al., 2019). Investigating these dual roles of TETs and dissecting their requirements in NPC biology and neurogenesis will be essential.

Although global or neural-specific loss of each *Tet* gene in mice influences NPC biology, it does not block neurogenesis or cause gross neuroanatomical defects (Rudenko et al., 2013; Zhang et al., 2013). However, combined loss of TET1/2 and TET1/3 causes exencephaly and holoprosencephaly in some embryos, respectively (Dawlaty et al., 2013; Kang et al., 2015) suggesting redundancy between TETs in neurogenesis that warrants further investigation. Findings from other organisms have also supported a role for TET enzymes in embryonic neurogenesis. *Xenopus laevis* embryos depleted of TET3 are microcephalic and eyeless with deregulation of neurodevelopmental programs leading to aberrant expression of neuronal, eye, neural crest, and sonic hedgehog signaling genes (Xu et al., 2012). Moreover, *tet2/3* mutant zebrafish exhibit abnormal brain and eye morphology (Li C. et al., 2015) and impaied retinal neurogenesis, partly due to overactive Notch and Wnt signaling (Seritrakul and Gross, 2017). Aberrant expression of mesodermal genes was also observed in *tet2/3* mutant retinas (Seritrakul and Gross, 2017), a finding similar to those in *Tet1/2/3* knockout embryos (Li et al., 2016). Importantly, TET enzymes mediate demethylation of conserved developmental enhancers in brain during the phylotypic stage of vertebrates, as demonstrated in zebrafish, *Xenopus tropicalis*, and mouse (Bogdanovic et al., 2016). Together, these findings support highly conserved and overlapping functions for TETs in neurodevelopment.

### Role of TET Enzymes and 5hmC in Postnatal Brain and Mature Neuronal Function

In addition to roles in regulation of NPC biology, TETs and 5hmC are important in postnatal neurodevelopment and mature neurons. As previously mentioned, 5hmC accumulates over the course of life (Szulwach et al., 2011). During development of mouse olfactory bulb neurons, which occurs throughout life, 5hmC is enriched in neurons relative to immature cells and is associated with increased neurodevelopmental gene expression (Colquitt et al., 2013). Likewise, 5hmC increases over the course of postnatal retinal maturation, and is enriched at neurogenesis genes (Perera et al., 2015). In the cerebellum, 5hmC increases during an important period of neuronal circuit formation, and TET1 and TET3 are required for proper branching of granule cell dendrites (Zhu et al., 2016). Chimeric *Tet3* knockout mice generated by injection of *Tet3* sgRNAs in one cell of a two-cell-stage embryo develop histologically normal cerebral cortices composed of *Tet3* wild type and knockout cells but exhibit abnormal electrophysiology in recordings of excitatory and inhibitory neurotransmission, suggesting that TET3 is required for developmental synapse and circuit formation (Wang et al., 2017). These studies implicate TETs in shaping the epigenetic landscape during specification of mature neural cell types after birth and in the development of higher order structures, including neuronal circuits.

5hmC and TET enzymes have also been shown to be highly dynamic within post-mitotic neurons. Cortical 5hmC has cell-type specific distributions associated with differential gene expression (Kozlenkov et al., 2018), and the ability of TETs to promote active DNA demethylation and alter gene expression in response to neuronal activity and to influence behavior has been the subject of extensive study (Guo et al., 2011; Kaas et al., 2013; Rudenko et al., 2013; Zhang et al., 2013; Li et al., 2014; Yu et al., 2015). *Tet1* expression is downregulated in response to neuronal activity in hippocampus where it regulates spatial memory and fear memory extinction (Guo et al., 2011; Kaas et al., 2013; Rudenko et al., 2013; Zhang et al., 2013). Hippocampal neurons upregulate *Tet3* to initiate active DNA demethylation in response to neuronal stimulation (Yu et al., 2015). In cortical neurons, *Tet3* is upregulated during fear extinction learning and, like *Tet1* in the hippocampus, is required for fear memory extinction (Li et al., 2014). Fear extinction learning is accompanied by *Tet3*-mediated upregulation of the *Gephyrin* gene and a transcriptionally-permissive reshaping of chromatin around this locus (Li et al., 2014). Loss of *Tet3* is sufficient to produce anxiety-like behaviors in mice, partly due to increased expression of immediate early genes like *Npas4* (Antunes et al., 2020), a role that is opposite to the anxiolytic and anti-depressant effects of *Tet1* (Feng et al., 2017). In general, the mechanistic basis by which TET enzymes influence behavior is, in part, due to reshaping of neuronal 5mC and 5hmC landscapes in response to activity. This remodeling of the epigenome is required for proper expression of genes involved in memory consolidation and synaptic function, such as *Bdnf* and *Arc*, and is sufficient to alter the electrophysiological properties of neurons (Guo et al., 2011; Kaas et al., 2013; Rudenko et al., 2013; Zhang et al., 2013; Li et al., 2014; Yu et al., 2015). In post-mitotic cerebellar neurons, 5hmC dynamics can influence recruitment of key gene regulatory factors. For example, 5hmC in gene bodies is associated with reduced MeCP2 occupancy and increased gene expression, possibly due to loss of MeCP2 repression (Mellen et al., 2017). Of note, reduced MeCP2 occupancy is specifically associated with 5hmC at gene body CpG dinucleotides, whereas 5hmC at CpA sites flanking enhancers retains MeCP2 binding. This highlights the ability of 5hmC to influence recruitment of gene regulatory factors in a sequence-dependent manner (Mellen et al., 2017). Moreover, findings that common *MECP2* mutations in Rett syndrome disrupt MeCP2 binding to 5hmC has implications for this mark in Rett syndrome pathogenesis (Mellen et al., 2012; Brown et al., 2016). Together, these studies propose crucial roles for 5hmC and TET enzymes in mature neuronal function.

### Implication of TET Enzymes and 5hmC in Human Neurodevelopmental and Neurodegenerative Disorders and Addiction

Compelling evidence for the importance of TETs in neurodevelopment and brain function is the identification of *TET* gene mutations and alterations in 5hmC levels in human neurodevelopmental and neurodegenerative disorders. Mutations in *TET3* were recently identified to underlie an inherited syndrome of intellectual disability and craniofacial abnormalities (Beck et al., 2020). While most mutations are in the catalytic domain and are sufficient to impair enzymatic activity, some are outside of this domain, and one mutation does not affect catalytic activity (Beck et al., 2020), underscoring the importance of TET3 catalytic and non-catalytic functions in human neurodevelopment. Interestingly, the clinical characteristics of patients with TET3 deficiency resemble those of patients with Tatton-Brown-Rahman syndrome and Sotos syndrome, caused by mutations in *DNMT3A* and *NSD1*, respectively (Kurotaki et al., 2002; Tatton-Brown et al., 2014). This highlights the general importance of DNA and histone methylation dynamics in human craniofacial and neural development. Other *TET* mutations have been observed in individuals with intellectual disability. Mutations in *TET1* were identified in consanguineous Pakistani and Iranian families with familial intellectual disability syndromes (Harripaul et al., 2018), and a germline *TET2* variant in an individual diagnosed with intellectual disability and delayed verbal comprehension in the absence of any other known genetic causes (Kaasinen et al., 2019). Together, these findings support an important role for TETs in the etiology of neurodevelopmental disorders and intellectual disability.

In addition TET enzymes and 5hmC are recurrently dysregulated in neurodegenerative conditions and in aging brain. Induced pluripotent stem cell-derived NPCs and neurons from Alzheimer’s disease (AD) patients exhibit differential hydroxymethylation at genes associated with neurodevelopment and synaptic function, including at known AD susceptibility loci, compared to cells derived from healthy controls (Fetahu et al., 2019). Consistently, presumptive loss-of-function mutations in *TET2* have been identified in patients with early onset AD and frontotemporal dementia (Cochran et al., 2020). Interestingly, TET2 promotes proinflammatory gene expression in microglia and *TET2* expression is increased in microglia associated with amyloid beta plaques in the brains of AD patients and mouse models (Carrillo-Jimenez et al., 2019). Thus, the positive and negative roles of TET2 in AD are likely specific to distinct stages in clinical course and cell types. *TET* variants or dysregulation have also been implicated in Parkinson’s disease (PD). *TET1* mutations were reported in a Chinese cohort of PD patients (Shu et al., 2019). Intriguingly, increased expression of *TET2* and increased 5hmC at neural enhancers is observed in prefrontal cortex of patients with PD and *Tet2* knockout mice are protected from inflammatory damage to the substantia nigra (Marshall et al., 2020). Conversely, *Tet2* expression declines in the hippocampus of aging mice and is associated with age-related cognitive decline (Gontier et al., 2018). Remarkably, restoration of hippocampal *Tet2* expression by stereotactic lentivirus injection is sufficient to improve cognitive function in aged mice (Gontier et al., 2018). Moreover, recent findings that TET1 and TET2 are required for axonal regeneration by reprogramming factor expression highlights their potential as therapeutic targets (Lu et al., 2020). These observations in human disease and mouse models warrant further studies to clarify the discordant roles of TET enzymes in the etiology of neurodegenerative diseases and aging.

In addition to their roles in neurodevelopmental and neurodegenerative disorders, TET enzymes are associated with addictive behaviors in humans. *TET1* expression is decreased in the nucleus accumbens (NAc) of humans suffering from cocaine addiction and cocaine administration to mice is sufficient to alter 5hmC at enhancers in NAc (Feng et al., 2015). *Tet* expression in NAc is also responsive to methamphetamine administration in rats (Jayanthi et al., 2018). Further work is necessary to clarify the role of TETs in mediating addictive behaviors.

## Discussion

A dozen years since the discovery that TET enzymes promote DNA hydroxylation and demethylation and the first studies reporting the abundance of 5hmC in the mammalian nervous system (Kriaucionis and Heintz, 2009; Tahiliani et al., 2009), work in the field has shed some light on their functions in neural physiology. TETs are dynamically expressed during development and in particular during embryonic and adult neurogenesis. 5hmC is a highly enriched mark in the brain and its levels increase over the course of embryonic neurogenesis and postnatal life where it is associated with neural gene expression. TET enzymes are required for various aspects of neurodevelopment, NPC biology, and neuronal activity. Findings that 5hmC and TETs are dysregulated in human neurodevelopmental and neurodegenerative disorders and addiction open new frontiers for utilizing them in clinical diagnostics and therapeutics. Despite this progress, several fundamental questions remain unanswered. These pertain to: (1) mechanisms of TET recruitment to target sites, (2) functional redundancy between TET paralogs, (3) gene activation and silencing by the dual enzymatic and non-enzymatic functions of TET enzymes, (4) relevance of 5hmC readers and interactomes of TETs in gene regulation, (5) re-establishment of 5hmC upon active DNA demethylation at activity-dependent genes in post-mitotic neurons, and (6) involvement of 5fC and 5caC, the other oxidized derivatives of 5mC, in DNA demethylation and beyond.

It has been substantiated that increases in gene body 5hmC is associated with activation of neural genes over the course of development, but the mechanism by which 5hmC influences gene expression remains incompletely understood. One possibility is that 5hmC recruits specific factors to promote transcription, and several groups have sought to identify such readers of 5hmC (Yildirim et al., 2011; Mellen et al., 2012; Spruijt et al., 2013). For example, MeCP2 binds 5hmC at neuronal genes to facilitate transcription, a finding with implications in the pathogenesis of Rett syndrome, where *MECP2* is mutated and 5hmC levels are altered (Mellen et al., 2012). Alternatively, evidence also supports a role for MeCP2 and MBD2 in protecting 5mC from conversion to 5hmC (Szulwach et al., 2011; Ludwig et al., 2017). In addition, UHRF2 is a specific reader of 5hmC in NPCs while THAP11 interacts with 5hmC in brain tissue (Spruijt et al., 2013). The functions of these and other 5hmC readers, including WDR76 and THY96, in the nervous system remain to be further explored (Spruijt et al., 2013). In contrast, the observation that elevated 5hmC levels may persist at genes after silencing suggests that any factor recruitment by this mark is not sufficient to maintain gene expression in the presence of antagonistic or in the absence of agonistic transcriptional cues (Colquitt et al., 2013). This would be consistent with a necessary but not sufficient role for TET-mediated 5hmC deposition and DNA demethylation for potent gene transcription (Baumann et al., 2019). This could also indicate a requirement for higher-order formation of 5fC and 5caC for transcription factor recruitment, given the fact that a growing number of proteins bind 5fC more specifically than 5hmC (Iurlaro et al., 2013). Additional work is needed to substantiate a causal role for 5hmC in neural gene regulation.

**FIGURE 1:**
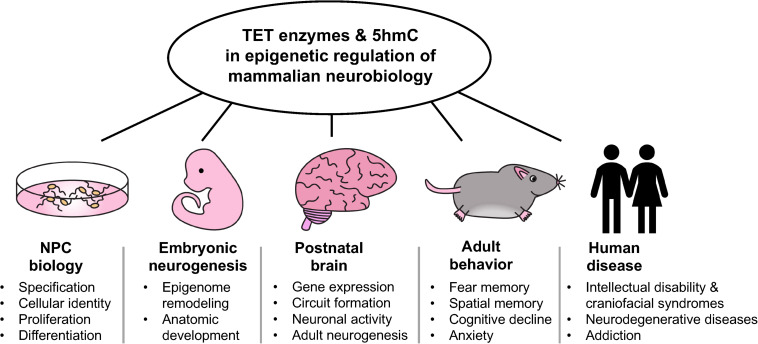
Overview of the multifaceted roles of TET enzymes and 5hmC in the epigenetic regulation of mamalian neurobiology.

Although functional studies have defined important roles for individual TETs in neural development and physiology, the absolute requirements of these enzymes and 5hmC is not fully established. This is in part due to the possible compensatory effects of TETs in studies involving deletion of individual *TET* genes. Leveraging conditional genetic systems for spatial and temporal deletion of all three *TET* genes in specific cell types may allow for identification of their novel functions in the brain. While TET enzymes certainly influence gene expression through their enzymatic functions, non-enzymatic activities of these proteins involving formation and recruitment of chromatin regulatory complexes have also been described (Chen et al., 2013; Ito et al., 2019; Montalban-Loro et al., 2019). Further work is necessary to fully elucidate protein-protein interactions by which TETs influence transcription and chromatin state, such as those between TET2 and FOXO3 in NPCs (Li et al., 2017) and TET3 and NSD3 in mature neurons (Perera et al., 2015). Comparison of the neural phenotypes associated with loss of TET enzymes vs. loss of their enzymatic activity alone will help dissect key enzymatic-dependent and independent functions of TET proteins in the brain. Use of existing and development of new *Tet* catalytic mutant mouse models will facilitate the *in vivo* study of TET catalytic-independent functions. Furthermore, the observation that loss of *Tet* in *Drosophila* results in aberrant brain development and reduced RNA hydroxymethylation warrants investigation into the role of TET-mediated RNA modifications in mammalian brains (Delatte et al., 2016). In summary, as illustrated in Figure 1, TET enzymes and 5hmC play crucial roles in various aspects of neurobiology, from regulation of NPCs and neurogenesis to adult brain function and human diseases. Gaining further insights into their roles will enhance our understanding of metazoan nervous system development and the etiology of human neurological disorders.

## Author Contributions

IM wrote the first draft and prepared the figure. MD edited and revised the draft and figure, and finalized the final version with input from IM. Both authors contributed to the article and approved the submitted version.

## Conflict of Interest

The authors declare that the research was conducted in the absence of any commercial or financial relationships that could be construed as a potential conflict of interest.
